# Validation of a Food Frequency Questionnaire for Assessing Fatty Acid Intake in Latvian Pregnant Women

**DOI:** 10.3390/nu17193108

**Published:** 2025-09-30

**Authors:** Ksenija Nikolajeva, Vinita Cauce, Laila Meija

**Affiliations:** 1 Riga East Clinical University Hospital, 2 Hipokrata Street, LV-1038 Riga, Latvia; laila.meija@rsu.lv; 2 Doctoral Department, Faculty of Medicine, Riga Stradiņš University, 16 Dzirciema Street, LV-1007 Riga, Latvia; 3 Faculty of Medicine, Riga Stradiņš University, 16 Dzirciema Street, LV-1007 Riga, Latvia; vinita.cauce@rsu.lv; 4 Department of Rehabilitation, Riga Stradiņš University, 26a Anninmuizas Boulevard, LV-1067 Riga, Latvia

**Keywords:** pregnancy, nutrition, fatty acids, validation, food frequency questionnaire

## Abstract

**Objectives**: During pregnancy, fat intake is crucial for fetal development and optimal outcomes, and validation instruments are essential for assessing dietary composition and nutrient intake. The aim of this research was to validate a food frequency questionnaire (FFQ) against a 7-day food record (FR) to measure fatty acid consumption during pregnancy. **Methods**: From July 2020 to June 2023, 138 women at 27–40 weeks’ gestation with a mean age of 31.5 ± 4.9 years were enrolled. Data were collected from medical records; an FFQ; a questionnaire gathering data on demographics, anthropometrics, health status, lifestyle, and use of food supplements at outpatient clinics; and a 7-day food record. Correlations between FA intakes from the FFQ and 7-day FR were assessed using Spearman’s rank-order correlation. **Results**: For the FFQ, correlation values ranged from 0.108 to 0.527, and all were statistically significant (*p* < 0.05) except for tetracosanoic acid. **Conclusions**: The developed FFQ is an accurate, valid instrument for assessing fatty acid (FA) intake among Latvian pregnant women and is reliable for future use in epidemiological studies.

## 1. Introduction

During gestation, women experience a series of physiological and anatomical adaptations that alter their nutritional demands [1]. Diet and lifestyle are key determinants of maternal and offspring health, beginning from the preconception period [2]. Maternal nutrition can modulate the risk of pregnancy-related complications, including alterations in blood pressure, gestational diabetes, and weight gain, while also impacting long-term chronic conditions and adverse outcomes for both the mother and fetus, which potentially extend into adulthood [3,4].

Lipids are essential nutrients that contribute to energy storage, tissue growth, and cell signaling, with the quantity and quality of fat in a pregnant woman’s diet playing a critical role in developmental programming and optimal fetal growth [5]. Numerous epidemiological studies have shown that fat intake often deviates from established nutritional guidelines [6,7,8].

The importance of maternal nutrition underscores the need to assess dietary intake during pregnancy, although this is complicated by factors such as cultural influences, belief systems, taboos, and physiological changes that alter dietary patterns throughout gestation [3,4]. Therefore, maternal socio-demographic characteristics and educational attainment may influence dietary patterns during pregnancy [9,10]. Further, the intake of supplements leads to modifications in the consumption of specific micronutrients [3]. Assessing dietary fatty acid (FA) intake is also complex due to substantial variability in FA profiles across food brands, changes in oil types used by manufacturers, and fluctuations in the species and proportions of fish oils incorporated, necessitating the use of consistent food composition data from the same reference period for accurate analysis [4]. Thus, accurate assessment of pregnant women’s food intake requires the use of specialized tools, developed and validated with rigorous methodological standards, to ensure precise measurement of dietary FA consumption within this population [3].

Various approaches are employed to evaluate the dietary intake of FA, including 24 h dietary recalls, food diaries, and food frequency questionnaires (FFQs) [11,12]. Additionally, certain validation studies may utilize specific biomarkers as reference measures [3]. Although food records (FRs) and 24 h dietary recalls can yield accurate dietary data, their implementation is challenging due to their complexity and the high level of participant compliance required [1,3]. In contrast, FFQs, instruments designed to quantitatively and qualitatively evaluate dietary intake within a specific population over a defined timeframe, are the most frequently used tools in epidemiological research as they offer a more comprehensive estimation of habitual dietary intake over an extended period [1,3]. An FFQ comprises a structured list of food items wherein respondents, depending on the FFQ type (quantitative, semi-quantitative, or qualitative), report their consumption frequency (e.g., daily, weekly, monthly, or annually) and portion sizes. The composition of the food list may be adapted according to the ethnic, social, and cultural characteristics of the target population [3]. FFQs are cost-effective and easily administered, making them a preferred method of dietary assessment in pregnancy-related studies [1,3,13]. FFQs require adaptation and validation for application within specific populations [1,3].

Validation of reliable instruments for assessing dietary composition and nutrient intake during pregnancy is essential. The validity of an FFQ should be assessed using multiple metrics and statistical methodologies. Valid FFQs are a critical component in the development of new dietary assessment tools, as inaccurate data can result in spurious associations between dietary factors and health outcomes or biomarkers [1,3]. As no definitive method for accurately measuring overall dietary intake exists, FFQ validation typically involves comparison with alternative self-reporting techniques, such as 24 h dietary recalls or FRs [4]. These measurements should consider the types of foods commonly consumed, their availability, traditional dietary practices and, in the case of pregnant individuals, the use of nutritional supplements [1]. Numerous semi-quantitative studies have used 24 h dietary recalls as a method for developing food lists [14,15,16,17]. Other approaches include an FR [18], use of seasonal food availability data [19,20], food composition databases [21], and expert consultation [3,22]. However, at the moment, none of these methods provide comprehensive data on the validation of detailed FA intake, highlighting a need to address this gap. Thus, the aim of this study was to evaluate the validity of an FFQ compared to a 7-day FR in assessing fat intake in pregnant women in Latvia.

## 2. Materials and Methods

### 2.1. Study Population

This cross-sectional study was carried out as part of the Latvian Council of Science project, titled “Excess Weight, Dietary Habits, and Vitamin D and Omega-3 Fatty Acid Status in Pregnancy” (Project No. Izp-2019/1-0335), which was conducted from July 2020 to June 2023. Quota sampling was used to stratify the target population by region based on the 2019 demographic data of women of reproductive age in Latvia, accounting for seasonal variations. The sample was formed by conducting surveys in outpatient institutions in Riga. Among 145 third-trimester pregnant participants, 138 women between 27 and 40 weeks of gestation who completed both the FFQ and 7-day food records were included in this study. Minor respondents, participants residing outside of Latvia, and those diagnosed with diabetes mellitus, gastrointestinal disorders (including inflammatory bowel diseases, short bowel syndrome, and celiac disease), or eating disorders were excluded from the study.

### 2.2. Data Collection

During the survey, two questionnaires were used: an FFQ and a questionnaire that gathered data on participant demographics, anthropometrics, health status, lifestyle, and use of food supplements. Anthropometric data and data on the course of pregnancy were obtained from medical records. In addition, the pregnant women had to complete a 7-day FR after the survey.

Both questionnaires and the food diary were available in Latvian and Russian. Before the start of the study, all researchers were trained at the Research Institute of Food Safety, Animal Health and Ecology “BIOR” to minimize errors.

#### 2.2.1. Food Frequency Questionnaire (FFQ)

The questionnaire recorded information on the participant’s diet over the past six months. It was adapted from a questionnaire used by the Scientific Institute of Food Safety, Animal Health and Environment “BIOR” to obtain data on nutrition among the Latvian population [23]. The questionnaire recorded the consumption frequency for a total of 211 items, of which 199 were food and beverages grouped into 20 product groups, including bread, pastries, breakfast cereals, jam, honey, creams, dairy products, fats and sauces, soups, meat products, prepared meat and vegetarian dishes, fish, eggs, potato dishes, pancakes and cottage cheese dishes, cereals, bread, flour, pasta, snacks, vegetables, fruits and berries, nuts and seeds, sweets, and beverages, and 12 items were food additives. To estimate the portion sizes of food and drinks, the “Photo Atlas of Food Products and Food Portions” was used.

#### 2.2.2. Seven-Day FR

Following the survey, the participants were asked to complete a 7-day FR. This diary was available in both paper and electronic form, and each participant’s FR was assigned a unique identification number. The participants were instructed to record the date and day of the week for each entry, specifying whether it was a weekday or a public holiday. Additionally, they were asked to indicate who had prepared the food they consumed that day.

The diary required detailed documentation of all foods, dishes, beverages, and food additives consumed over the previous 24 h for each of the seven consecutive days. For each item, the participants were asked to report the name of the food, the time of consumption, the portion size (in grams or units), and whether the item was home-prepared, commercially catered, or industrially manufactured. In cases involving self-prepared or collected foods, the individual ingredients needed to be listed separately. For processed or ready-to-eat items, the participants were also encouraged to note the manufacturer, if known.

To support accurate and consistent reporting, the participants received training during the interview process and were provided with a specially developed food atlas designed to minimize reporting errors.

### 2.3. Ethics

Ethical approval was obtained from the Clinical Research Ethics Committee of Riga Stradiņš University (No. 6-1/02/62) for this study.

Before the survey, the study participants were provided with written information about the study’s objectives and purposes. After signing a consent form to participate in the study, the participants were assigned a code number so their data could be recorded and processed.

### 2.4. Data Analysis

Dietary data obtained from the FFQ and 7-day FR were processed at the Institute of Food Safety, Animal Health and Environment “BIOR” using a custom software developed on the Microsoft Dynamics Ax 2009 platform. This software integrates the BIOR Food Composition Database, which was originally established for the “Food Consumption Study of the Latvian Population (2012–2013)” and has since been continuously updated and utilized in subsequent national dietary surveys. The database is based on the food composition data from the German Max Rubner Institute and supplemented with information on Latvian-specific food products and traditional recipes. The analysis of dietary records provided estimates of total energy intake, macro- and micronutrient consumption, and the intake of individual FAs. This study involved pregnant women who completed an FFQ, a 7-day FR, and whose daily caloric intake ranged between 600 and 4000 kcal.

Statistical analysis was performed using SPSS software, version 29.0. Descriptive statistics—including measures of central tendency and dispersion—were applied to summarize the data. Categorical variables are expressed as frequencies and percentages, while continuous variables are reported as either means with standard deviations or medians with interquartile ranges, depending on the distribution of the data. The correlation between FAs reported using the FFQ and 7-day FR was evaluated using Spearman’s rank-order correlation. Statistical significance was established at a *p*-value threshold of <0.05.

## 3. Results

### 3.1. Baseline Characteristics

Table 1 summarizes the baseline characteristics of the participants enrolled in this study, which included 138 women in their 27th–39th week of pregnancy. The participants had a mean age of 31.5 ± 4.9 years and were predominantly married Latvian women with a normal body mass index (BMI) and a higher education. A total of 19.2% (n = 25) women were classified as overweight or obese before pregnancy.

Lifestyle data, including daily exercise during pregnancy, alcohol consumption, and smoking, are shown in Table 2. Most women abstained from alcohol during pregnancy but, among those who did consume it, the majority did so during the first trimester, as reported by 76% of respondents (n = 38).

### 3.2. Dietary Intake

The daily energy and macronutrient intake recorded by the FFQ and 7-day FR are shown in Table 3. The FFQ indicated higher energy and macronutrient intakes.

Statistically significant correlations (Table 4 and Table 5) were observed for saturated fatty acids (SFAs), monounsaturated fatty acids (MUFAs), and polyunsaturated fatty acids (PUFAs), with Spearman correlation coefficients of 0.296, 0.188, and 0.354, respectively, as well as for all FAs with a Spearman coefficient ranging from 0.209 for C20:0 to 0.527 for C20:5 (*n*-3). C24:0 from the FFQ demonstrated a lower correlation and did not reach statistical significance.

## 4. Discussion

The objective of our study was to assess the validity of an FFQ in comparison with a 7-day FR for measuring the intake of fatty acids among pregnant women. Based on reference values from Ortiz-Andrellucchi et al., who described correlation values for assessing the quality of a dietary intake tool—considering a correlation <0.30 to be poor, 0.30–0.50 to be acceptable, 0.51–0.70 to be good, and >0.70 to be very good—our study showed poor and acceptable correlations between all FA groups and FAs recorded in the food diary and FFQ. All were statistically significant except for tetracosanoic acid (C24:0) [24,25]. A good correlation coefficient was found for C20:5 (eicosapentaenoic acid, EPA).

Most previous studies focused on assessing the overall diet of pregnant women and validating nutrients, vitamins, and minerals. Only a few provided a more detailed comparison of SFAs, MUFAs, and PUFAs. In a study intended to develop a semi-quantitative FFQ targeting the Mediterranean culture and evaluating its accuracy, the validity of the FFQ was assessed by comparing it with 24 h dietary records in pregnant women (n = 179). The coefficients found for SFs, MUFAs, and PUFAs were 0.77, 0.55, and 0.54. For pregnant women in Northeastern Brazil (n = 100), correlation coefficients between an applied FFQ and the mean of three 24 h records for SFAs, MUFAs, and PUFAs were 0.76, 0.87, and 0.73, respectively (*p* < 0.001) [17]. Lower but still statistically significant values were found in Almulla et al.’s study—0.19, 0.2, and 0.2 for SFAs, MUFAs, and PUFAs—which aligned more closely with our results [26].

No existing FFQ has assessed FAs in detail, and our study is the first to include such a wide range of FAs (n = 33). Most previous studies concentrated on *n*-3 long-chained PUFAs (LC-PUFAs) [27,28,29]. In Indonesian pregnant women (n = 100) in the third trimester, the correlation coefficients observed between a semi-quantitative FFQ and a 2-day-record (2DR) were 0.38, 0.34, and 0.35, for EPA, docosahexaenoic acid (DHA), alpha-linolenic acid (ALA), and total *n*-3, respectively. These values are lower than those in our study (0.527, 0.467, and 0.373, respectively) [29]. Similar correlation coefficients were found in [27] for LC-PUFA intake from an FFQ estimated using dietary records, with values ranging from 0.34 to 0.4, *p* < 0.0001.

Differences in values can be explained by the peculiarities of FA metabolism during pregnancy. Correlation and statistical significance may also be related to the ease of assessing portion sizes, day-to-day variability in dietary intake, and the absence of an interval between the completion of the FFQ and the 7-day FR. Since foods high in eicosapentaenoic and docosahexaenoic acids, like fish and seafood, are not consumed regularly, the FFQ is beneficial because it evaluates dietary intake over a longer time frame [24].

Most studies have a varying number of items, ranging from 46 to 221 (121 items on average). The greater number of items in our study (199 items) is associated with higher correlation coefficients compared to short FFQs, although according to the literature, it is also associated with increased heterogeneity in validation studies [3].

A limitation of this study is that the reproducibility of the FFQ was not investigated and the long-term reliability of the FFQ cannot be substantiated. Additionally, the validity of the FFQ was only evaluated in a healthy population. Completing the FR and FFQ could have also introduced method-inherent limitations associated with memory bias, as well as difficulty participating for the interviewees, depending on their ability and psychological condition [17,25]. FFQs may overestimate intake of certain food groups, particularly under-consumed items like vegetables, and often do not capture absolute quantities. Estimates are thought to reflect habitual dietary patterns and perceptions rather than precise memory of specific intake events [30,31]. Longer FR assessments can be associated with decreased participant motivation, potentially reducing data accuracy [12]. However, interview-administered visual examples of food portions from the Food Product Atlas, detailed descriptions, and the possibility to contact researchers while completing dietary records were implemented to minimize these limitations. Our study was performed by trained professionals, which greatly enhances the accuracy of reporting [12]. Moreover, the use of 7-day FR for separate days of the week (weekdays and weekend days) instead of 24 h recall to validate the FFQ may have helped improve the relative validity of the FFQ by providing better estimates of nutrient intake [32]. As the study was conducted in Latvia, the applicability of these findings to other cultural or dietary contexts is limited, and therefore the study has limited external validity. Future studies should focus on validating the FFQ in different regions. The development of the validation study and the design of the developed instrument, as well as the reliability of the reference method data, could also affect the correlation coefficients [3].

Some validation studies have used blood biomarkers as reference for dietary evaluation [22,29]. However, hormonal fluctuations during pregnancy induce significant changes in lipid metabolism. Although these physiological and hormonal adaptations do not directly impair lipid metabolism, they may result in notable shifts in fatty acid profiles [18]. Consequently, FFQs may offer a more accurate representation of maternal dietary patterns during gestation and are recommended for use in nutritional assessment in this population.

Our study showed that the FFQ investigated can be used for detailed evaluations of FA consumption, although correlations should be interpreted with caution. It may also be suitable for use in epidemiological studies due to its ease of administration and low cost. Additional research could enhance our understanding of fatty acid intake and facilitate its association with health outcomes. This FFQ may serve as a valuable tool for exploring the association between dietary intake and health outcomes, as well as identifying dietary factors influencing nutritional status [3].

## 5. Conclusions

Overall, the FFQ demonstrated poor and acceptable statistically significant correlations with the 7-day FR method. The developed FFQ provides a valid and reliable measure of fatty acid intake in Latvian pregnant women and is appropriate for population-level use. This instrument could be considered as reliable for future use in epidemiological studies, although generalizability to other cultural or dietary contexts is constrained. Future reproducibility studies and an evaluation of correlations with relevant biomarkers are recommended.

## Figures and Tables

**Table 1:** Participants’ baseline characteristics (n = 138).

Characteristic	*n* (%)
Nationality	
Latvian	81 (65.3)
Russian	42 (33.9)
Other	1 (0.8)
Marital status	
Married	105 (77.8)
Live in partnership	29 (21.5)
Single mother	1 (0.7)
Education	
Primary/incomplete secondary education	3 (2.2)
General special/secondary education	13 (9.7)
College education	4 (3.0)
Higher education	105 (78.4)
Incomplete higher education	9 (6.7)
BMI before pregnancy	
<18.5	10 (7.7)
18.5–24.9	95 (73.1)
25.0–29.9	21 (16.1)
≥30.0	4 (3.1)

BMI—body mass index, kg/m^2^.

**Table 2:** Physical activity, alcohol use, and smoking during pregnancy (n = 138).

n (%)	Variable
	Frequency of at least 30 min of physical exercise during pregnancy
2 (1.5)	Daily
9 (6.6)	4–6 times a week
50 (36.8)	2–3 times a week
24 (17.6)	Once a week
6 (4.4)	2–3 times a month
42 (30.9)	Some occurrences annually or less frequently or never
3 (2.2)	I cannot exercise due to illness or disability
	Alcohol consumption during pregnancy
22 (16.5)	Yes
83 (62.4)	No
28 (21.1)	I had not consumed it after I found out about the current pregnancy
	Smoking during pregnancy
2 (1.5)	Yes
74 (56.5)	No
1 (0.8)	Yes, but quit at one week of pregnancy
54 (41.2)	I have never smoked

**Table 3:** Daily intake of energy and nutrients from food and supplements (n = 138).

Median (Q1–Q3)		Energy/Nutrient
7-Day FR	FFQ	
2040.7 (1801.9–2403.6)	2328.3 (1788.7–2847.6)	Energy (kcal)
87.3 (73.3–99.0)	102.5 (76.8–141.1)	Protein (g)
207.8 (178.0–257.6)	221.9 (167.6–277.2)	Carbohydrate (g)
93.2 (81.3–108.9)	107.8 (77.2–135.5)	Fat (g)

FFQ—food frequency questionnaire; FR—food record; Q1–Q3—Quartile 1–Quartile 3.

**Table 4:** Mean daily intake of FA groups and Spearman correlation coefficients between FA groups from FFQ and 7-day FR.

*p* Value	r	Mean ± SD		FA Group
		7-Day FR	FFQ	
0.01	0.296	33.2 ± 10.7	37.5 ± 15.3	SFA
0.05	0.188	31.7 ± 9.6	41.6 ± 18.0	MUFA
0.01	0.354	13.2 ± 5.3	16.4 ± 8.5	PUFA

FA—fatty acid; FFQ—food frequency questionnaire; FR—food record; SFA—saturated fatty acid; MUFA—monounsaturated fatty acid; PUFA—polyunsaturated fatty acid; r—Spearman correlation coefficient.

**Table 5:** Daily intake of FAs and Spearman correlation coefficients between FAs recorded in FFQ and 7-day FR.

FA Group	FA	Median (Q1–Q3)		r	*p* Value
		FFQ	7-Day FR		
SFA	C4:0	1.03 (0.63–1.32)	1.01 (0.76–1.35)	0.397	<0.001
	C6:0	0.66 (0.40–0.84)	0.64 (0.49–0.86)	0.399	<0.001
	C8:0	0.43 (0.28–0.57)	0.52 (0.35–0.68)	0.420	<0.001
	C10:0	0.88 (0.58–1.14)	0.91 (0.66–1.19)	0.419	<0.001
	C12:0	1.24 (0.90–1.67)	1.36 (0.99–1.89)	0.376	<0.001
	C14:0	3.83 (2.56–4.84)	3.81 (2.77–4.90)	0.367	<0.001
	C15:0	0.38 (0.25–0.49)	0.36 (0.25–0.44)	0.358	<0.001
	C16:0	19.35 (13.47–24.14)	16.74 (13.01–29.05)	0.280	<0.001
	C17:0	0.35 (0.23–0.44)	0.31 (0.22–0.38)	0.299	<0.001
	C18:0	7.68 (5.29–9.89)	6.63 (5.02–7.93)	0.272	<0.001
	C20:0	0.37 (0.26–0.46)	0.30 (0.25–0.38)	0.209	<0.050
	C24:0	0.02 (0.01–0.04)	0.01 (0.01–0.03)	0.108	0.208
MUFA	C14:1	0.47 (0.32–0.63)	0.45 (0.32–0.56)	0.314	<0.001
	C15:1	0.20 (0.12–0.26)	0.19 (0.13–0.24)	0.390	<0.001
	C16:1	2.14 (1.60–2.80)	1.90 (1.49–2.29)	0.256	<0.001
	C17:1	0.32 (0.21–0.39)	0.30 (0.22–0.38)	0.368	<0.001
	C18:1 (*n*-9)	24.32 (26.59–44.10)	27.31 (22.52–32.45)	0.187	<0.050
	C20:1 (*n*-9)	0.50 (0.36–0.67)	0.41 (0.30–0.54)	0.310	<0.001
	C22:1 (*n*-9)	0.19 (0.13–0.30)	0.05 (0.03–0.10)	0.249	<0.001
PUFA	C18:3 (*n*-3)	2.47 (1.61–3.50)	1.66 (1.28–2.26)	0.373	<0.001
	C18:4 (*n*-3)	0.01 (0.00–0.02)	0.00 (0.00–0.02)	0.341	<0.001
	C20:5 (*n*-3)	0.21 (0.10–0.47)	0.15 (0.04–0.32)	0.527	<0.001
	C22:5 (*n*-3)	0.02 (0.01–0.04)	0.02 (0.01–0.05)	0.297	<0.001
	C22:6 (*n*-3)	0.33 (0.17–0.56)	0.23 (0.11–0.43)	0.467	<0.001
	C24:2 (*n*-3)	<0.01	<0.01	0.284	<0.001
	C18:2 (*n*-6)	13.64 (9.73–18.79)	9.44 (7.59–12.05)	0.382	<0.001
	C20:2 (*n*-6)	0.03 (0.02–0.05)	0.02 (0.01–0.03)	0.328	<0.001
	C20:3 (*n*-6)	0.02 (0.02–0.05)	0.02 (0.01–0.03)	0.327	<0.001
	C20:4 (*n*-6)	0.21 (0.16–0.32)	0.20 (0.14–0.30)	0.302	<0.001
	C22:3 (*n*-6)	<0.01	<0.01	0.301	<0.001
	C22:4 (*n*-6)	<0.01	<0.01	0.299	<0.001

FA—fatty acid; FFQ—food frequency questionnaire; SFA—saturated fatty acid; MUFA—monosaturated fatty acid; PUFA—polyunsaturated fatty acid; Q1–Q3—Quartile 1–Quartile 3; r—Spearman correlation coefficient.

## Data Availability

The data presented in this study are available upon request from the corresponding author.

## Abbreviations

The following abbreviations are used in this manuscript:

ALA	Alpha-linolenic acid
BMI	Body mass index
DHA	Docosahexaenoic acid
EPA	Eicosapentaenoic acid
FA	Fatty acid
FFQ	Food frequency questionnaire
FR	Food record
LC-PUFA	Long-chained polyunsaturated acid
MUFA	Monounsaturated fatty acid
PUFA	Polyunsaturated fatty acid
SFA	Saturated fatty acid
